# Prescription Rates, Polypharmacy and Prescriber Variability in Swiss General Practice—A Cross-Sectional Database Study

**DOI:** 10.3389/fphar.2022.832994

**Published:** 2022-02-14

**Authors:** Yael Rachamin, Levy Jäger, Rahel Meier, Thomas Grischott, Oliver Senn, Jakob M. Burgstaller, Stefan Markun

**Affiliations:** Institute of Primary Care, University of Zurich and University Hospital Zurich, Zurich, Switzerland

**Keywords:** drug prescriptions, polypharmacy, clinical practice variation, demographic aging, sex differences, primary care, Switzerland

## Abstract

**Purpose:** The frequency of medication prescribing and polypharmacy has increased in recent years in different settings, including Swiss general practice. We aimed to describe patient age- and sex-specific rates of polypharmacy and of prescriptions of the most frequent medication classes, and to explore practitioner variability in prescribing.

**Methods:** Retrospective cross-sectional study based on anonymized electronic medical records data of 111 811 adult patients presenting to 116 Swiss general practitioners in 2019. We used mixed-effects regression analyses to assess the association of patient age and sex with polypharmacy (≥5 medications) and with the prescription of specific medication classes (second level of the Anatomical Therapeutic Chemical Classification System). Practitioner variability was quantified in terms of the random effects distributions.

**Results:** The prevalence of polypharmacy increased with age from 6.4% among patients aged 18–40 years to 19.7% (41–64 years), 45.3% (65–80 years), and 64.6% (81–92 years), and was higher in women than in men, particularly at younger ages. The most frequently prescribed medication classes were *antiinflammatory and antirheumatic products* (21.6% of patients), *agents acting on the renin-angiotensin system* (19.9%), *analgesics* (18.7%), and *drugs for acid related disorders* (18.3%). Men were more often prescribed agents targeting the cardiovascular system, whereas most other medications were more often prescribed to women. The highest practitioner variabilities were observed for *vitamins*, for *antiinflammatory and antirheumatic products,* and for *mineral supplements*.

**Conclusion:** Based on practitioner variability, prevalence, and risk potential, antiinflammatory drugs and polypharmacy in older patients appear to be the most pressing issues in current drug prescribing routines.

## Introduction

A global increase in life expectancy has been observed in recent decades (GBD 2017 Mortality Collaborators, 2018), resulting in an older and more chronically ill population (Barnett et al., 2012; Cao et al., 2020). This demographic change is inevitably accompanied by an increasing need for medical interventions such as medication prescribing. Accordingly, the prevalence of polypharmacy (i.e., concurrent prescription of five or more medications) has climbed to over 25% among the older population in many healthcare systems (Guthrie et al., 2015a; Midão et al., 2018; Khezrian et al., 2020). This phenomenon is concerning because incremental health benefits tend to decrease with each additional medication, while the risk of adverse effects increases and may even outweigh the expected benefits (Kongkaew et al., 2013; Donaldson et al., 2017; Insani et al., 2021). The risk associated with prescribing varies greatly among different medication classes, with some medications (e.g., vitamins) posing minimal risks and others (e.g., anti-inflammatory drugs) posing substantial risks (Singh and Triadafilopoulos, 1999; McGettigan and Henry, 2011).

Given the potential negative health consequences of excessive prescribing and, in particular, polypharmacy, unwarranted variability in prescribing is of particular concern. Prescribing variability is unwarranted when it depends on physician factors (i.e., recognition of an indication) rather than patient factors (i.e., the presence of an indication) (Wennberg, 2011). Practitioner variability can thus serve as an indicator of issues with indication quality and potential healthcare inequity, which are particularly problematic in publicly funded healthcare systems like the Swiss.

Large primary care databases have been used before for measuring prescription rates and general practitioner (GP) variability in prescribing, but analyses have generally been limited to specific medication classes (e.g., antibiotics or opioids) (Guthrie et al., 2015b; Haastrup et al., 2016; Coyle et al., 2019) or populations (e.g., older patients) (Aubert et al., 2016; Schnegg et al., 2020). Comprehensive assessments across all medication classes and patient demographics are needed to identify the specific medication classes contributing to polypharmacy and to develop targeted initiatives to improve prescribing practices.

Therefore, the aim of the present study was to comprehensively describe medication prescribing in Swiss general practice and, in particular, to present patient age- and sex-specific prescription rates as well as practitioner variability in polypharmacy and the most common medication classes.

## Methods

### Study Design, Setting, and Participants

We performed a retrospective cross-sectional study based on data from the large Swiss primary care database FIRE (FIRE is an acronym for Family Medicine ICPC Research using Electronic Medical Records) (Chmiel et al., 2011). Since the FIRE project started in 2009, over 700 individual GPs have voluntarily contributed anonymized clinical routine data from their electronic medical records to the FIRE database (>10% of all Swiss GPs (mfe Haus- und Kinderärzte Schweiz, 2020)). As of April 2021, the database holds over 11 million consultation records with administrative information, laboratory and vital signs measures as well as medication plans.

For this study, we included GPs of practices exporting medication data labelled with starting and stopping dates since at least the year 2018 and covering the full year 2019 (26.6% of FIRE practices in 2019). From included GPs, we considered all patients aged 18 years or older who had at least one consultation in the year 2019 (total number of considered patients: *n* = 112 934). We grouped patients of the same sex and age (in years) into sex × age strata and excluded all patients in strata of less than 100 patients (i.e., all patients aged >92 years, *n* = 1 123). This left 111 811 patients in 150 sex × age strata for analysis.

The local Ethics Committee of the Canton of Zurich waived approval for the present study because the FIRE project is outside the scope of the law on human research (BASEC-Nr. Req-2017–00797).

### Database Query and Definitions

We extracted GP- and patient-level data from the database. From GPs, we used sex and year of birth. From patients, we used sex, year of birth, and the list of active medication prescriptions at their last consultation in 2019. We labeled medication prescriptions with the anatomical therapeutic chemical (ATC) classification system (WHOCC, 2020). The ATC classification system organizes active substances in a hierarchy with five different levels, according to the organ or system on which they act and their therapeutic, pharmacological, and chemical properties. We identified ATC codes of all recorded prescriptions, and excluded non-systemic medications, namely: 1) dermatologicals (ATC code D) except antifungals for systemic use (D01B), antipsoriatics for systemic use (D05B), and anti-acne preparations for systemic use (D10B); 2) drugs targeting sensory organs (S), i.e., ophthalmologicals and otologicals; 3) stomatological preparations (A01); 4) antiinflammatory preparations for topical use (M02AA); 5) throat preparations (R02); 6) agents for treatment of hemorrhoids and anal fissures for topical use (C05A); 7) decongestants and other nasal preparations for topical use (R01A); and 8) heparins or heparinoids for topical use (C05BA). Lastly, we excluded vaccines (J02) because of inconsistent data capturing.

For each patient, the medication count was defined as the number of distinct, active ATC codes (considering all five levels of the code), and polypharmacy was defined as a medication count ≥5. For the remaining analyses, prescriptions were aggregated on the second level of the ATC code, which represents therapeutic or pharmacological subgroups. We referred to the aggregated prescriptions as “(medication) classes”. We adopted the names of the classes as defined in the ATC classification system.

### Statistical Analysis

We used counts and proportions (*n* and %) or medians with interquartile ranges (IQRs) to describe the data. Prescription rates and polypharmacy rates per stratum (overall, sex-specific, age-specific, sex × age-specific) were calculated as proportions of patients with a prescription within a specific medication class or with polypharmacy, respectively. Sex × age-specific prescription rates of the different medication classes as well as sex-specific and age-specific rates of polypharmacy were presented graphically.

For statistical modelling, patient age was categorized into the groups 18–40 years, 41–64 years, 65–80 years, and 81–92 years. For each medication class separately, we built a mixed-effects logistic regression model of whether a patient had a matching prescription, with demographic patient variables as fixed effects (age groups and sex, with interaction terms) and random GP effects. An analogous model was built for the presence of polypharmacy. For the regression analyses, only GPs with at least 500 patients in 2019 were considered (*n* = 100 of total *n* = 116).

To explore practitioner variability in prescribing, we reported crude distributions of GP-specific prescription rates (again considering only GPs with at least 500 patients). Also, using the 5th and 95th percentiles of the fitted random effects distributions, we quantified the variability unexplained by covariates, both of prescribing the various medication classes and of polypharmacy, using the odds ratios OR_lib/cons_ = exp (*q*_0.95_ - *q*_0.05_), which are interpreted as the odds ratio (OR) of prescribing the respective medication class or of polypharmacy, respectively, between a rather liberal prescriber (at the 95th percentile of the random effects distribution) and a rather conservative prescriber (at the 5th percentile of the random effects distribution) for a patient of the same sex and age group. We reported results for the 20 medication classes with the highest overall prescription rates in the main text (out of a total of 72 appearing in the database); the appendix expands results to all classes with overall prescription rates >1%. For statistical analysis and visualization we used the R software (R Core Team. R, 2019), Version 4.0.0. Significance was assumed for *p*-values < 0.005 (Benjamin et al., 2018); 99.5% confidence intervals (CIs) were reported.

## Results

### Patient and GP Population

We analyzed 111 811 adult patients of 116 different GPs. Patients are described in Table 1, overall and by age group (for description of patients by sex, see Supplementary Table S1). Of GPs, 34.5% were female, the median GP age was 52 years (IQR 43–57), and GPs treated 923 patients (IQR 643–1 218) in median.

**TABLE 1:** Description of patients overall and by age group.

Variables	Overall (*n* = 111 811)	Age 18–40 years (*n* = 36 458)	Age 41–64 years (*n* = 44 167)	Age 65–80 years (*n* = 22 698)	Age 81–92 years (*n* = 8 488)
Female sex, %	51.7	52.0	49.9	51.7	60.2
Age, median (IQR)	52 (35–66)	30 (24–35)	53 (47–58)	72 (68–76)	85 (83–88)
Number of consultations in 2019, median (IQR)	4 (2–9)	2 (1–5)	4 (2–8)	7 (3–13)	10 (4–19)
Medication count, median (IQR)	2 (0–4)	1 (0–2)	2 (0–4)	4 (2–7)	6 (3–10)
Prevalence of polypharmacy (≥5 medications), %	24.0	6.4	19.7	45.3	64.6
Prescription rates of medication classes, %					
Antiinflammatory and antirheumatic products	21.6	16.5	23.9	26.3	18.6
Agents acting on the renin-angiotensin system	19.9	1.3	18.2	42.4	47.8
Analgesics	18.7	11.3	17.3	24.3	43.4
Drugs for acid related disorders	18.3	7.9	18.4	29.4	33.3
Vitamins	15.5	6.9	14.0	24.7	35.4
Antithrombotic agents	14.2	1.2	8.5	32.3	51.5
Lipid-modifying agents	12.1	0.3	9.7	30.8	25.9
Beta blocking agents	10.5	1.0	7.5	23.2	33.4
Psychoanaleptics	10.5	6.2	11.2	12.3	20.3
Psycholeptics	9.3	3.1	8.1	14.7	27.5
Mineral supplements	8.8	4.2	7.6	14.6	20.1
Drugs for obstructive airway diseases	8.7	6.8	8.5	11.4	11.0
Antianemic preparations	8.3	6.9	6.9	10.0	17.9
Drugs for constipation	6.7	2.5	4.7	10.8	24.1
Antihistamines for systemic use	6.0	6.7	6.1	5.1	4.7
Calcium channel blockers	5.5	0.3	3.9	11.7	19.0
Diuretics	5.1	0.1	2.1	10.4	28.2
Drugs used in diabetes	5.0	0.5	4.5	10.9	11.4
Urologicals	5.0	0.6	3.8	11.3	12.6
Thyroid therapy	4.4	1.9	4.3	6.9	9.1

Abbreviations: IQR, interquartile range.

### Medication Counts and Polypharmacy

**FIGURE 1:**
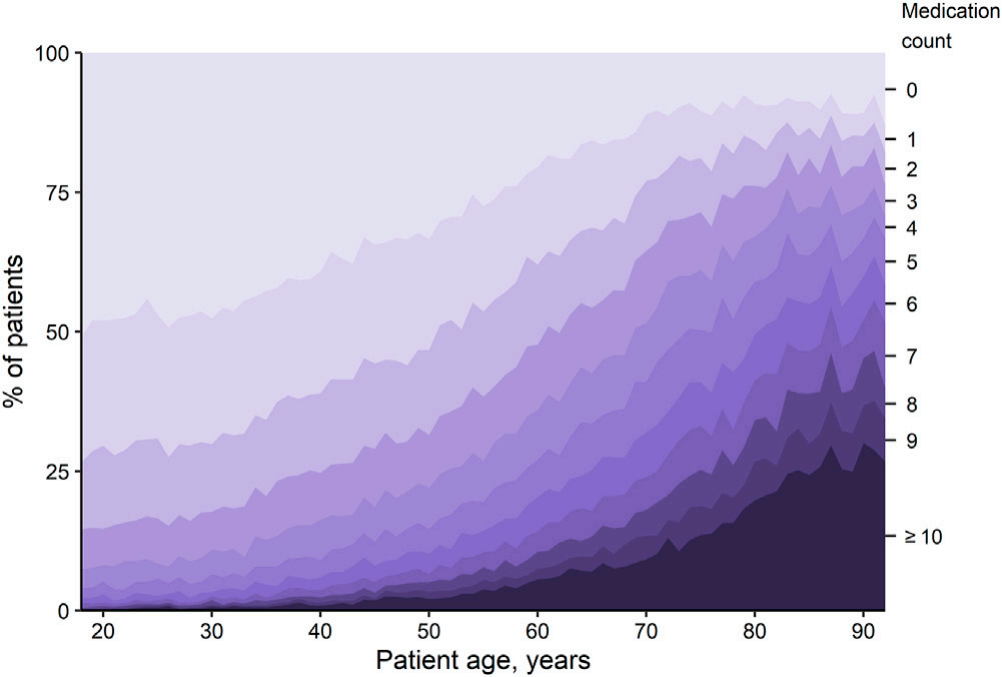
Proportion of patients with the specified number of medications, depending on age. Each shaded region represents the overall percentage of patients with the respective number of medications.

**FIGURE 2:**
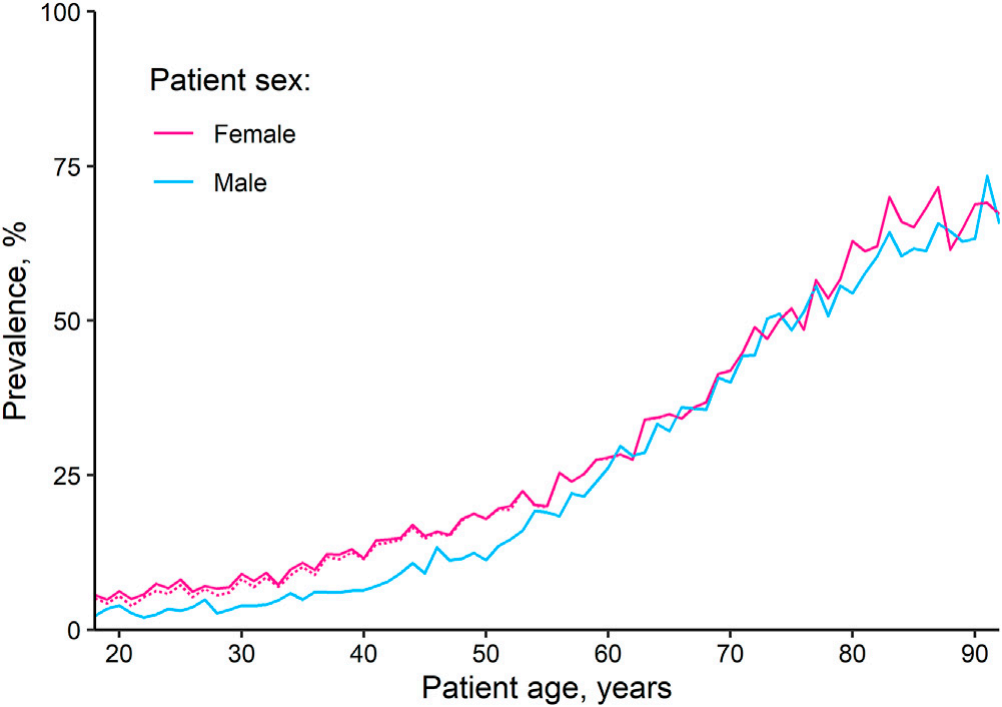
Age-dependent polypharmacy rates, by sex. The dotted line represents the proportion of female patients with polypharmacy if hormonal contraceptives for systemic use are excluded.

Overall, 28.9% of patients were without medication, 47.2% were prescribed one to four medications, and 24.0% had polypharmacy. The median medication count was 2 (IQR 0–4) overall and 3 (IQR 2–6) among patients with at least one medication. Medication counts (Figure 1) and, as a consequence, the likelihood of having polypharmacy (Figure 2), increased with patient age (see Table 1 for crude numbers and Supplementary Table S2 for regression analyses). Male patients exhibited considerably lower rates of polypharmacy than female patients in all age groups except 65–80 years (age 18–40 years: OR = 0.49 [99.5% CI 0.43 to 0.55], age 41–64 years: OR = 0.78 [99.5% CI 0.73 to 0.84], age 81–92 years: OR = 0.82 [99.5% CI 0.72 to 0.94], Supplementary Table S2). The difference remained even after hormonal contraceptives were excluded (Figure 2).

### Medication Classes

The most often prescribed medication classes were *antiinflammatory and antirheumatic products* (prescribed for 21.6% of patients), *agents acting on the renin-angiotensin system* (19.9%), *analgesics* (18.7%), and *drugs for acid related disorders* (18.3%; see Supplementary Table S3 for all prescription rates >1%).

**FIGURE 3:**
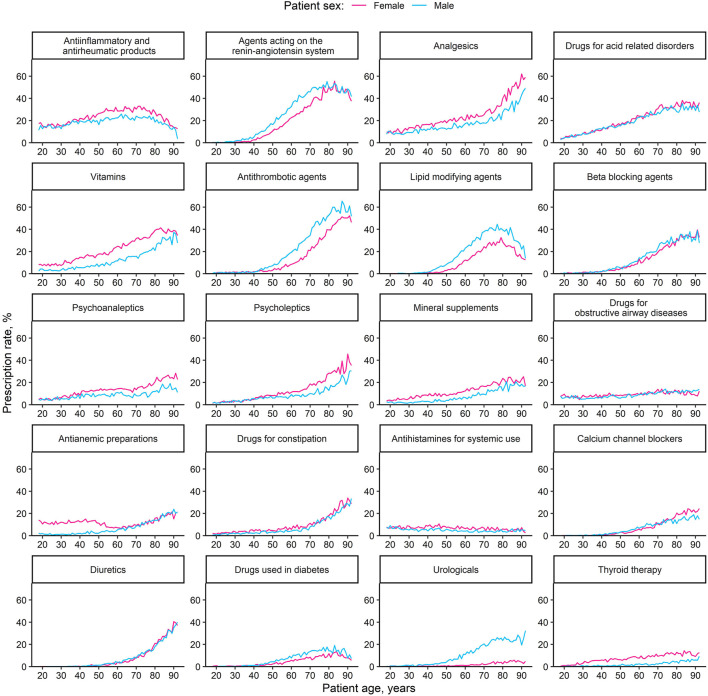
Age-dependent prescription rates of different medication classes, by sex. The 20 most common medication classes (second level of the anatomical therapeutic chemical classification system) are displayed, ordered by decreasing overall prescription rate.

For the vast majority of medication classes, prescription rates significantly increased with age (Figure 3; Supplementary Table S2 for regression models). Exceptions were *antihistamines*, which showed a decrease with age, *antianemic preparations*, which decreased in female patients at middle ages before increasing again at older ages, and *antiinflammatory and antirheumatic products* as well as *lipid modifying agents*, which increased up to a certain age before decreasing.

Prescription rates of all medication classes showed significant sex differences at certain ages (Figure 3 and Supplementary Table S2). Men were more often prescribed agents targeting the cardiovascular system (such as *antihypertensive agents*, *antithrombotic agents*, and *lipid modifying agents*), whereas most remaining classes were more often prescribed to women. Consistently significant and unidirectional prescription rate differences across all age groups appeared in *antiinflammatory and antirheumatic products* (female > male), *analgesics* (female > male), *vitamins* (female > male), *lipid modifying agents* (male > female), *psychoanaleptics* (female > male), *mineral supplements* (female > male), *drugs for constipation* (female > male), *urologicals* (male > female), and *thyroid therapy* (female > male). Some medication classes exhibited sex differences only in older ages (*drugs for acid related disorders*, *drugs used in diabetes*), while for others, sex differences existed at younger ages and diminished with age (*drugs for obstructive airway disease*, *antianemic preparations*, and *antihistamines for systemic use*).

### Practitioner Variability

**FIGURE 4:**
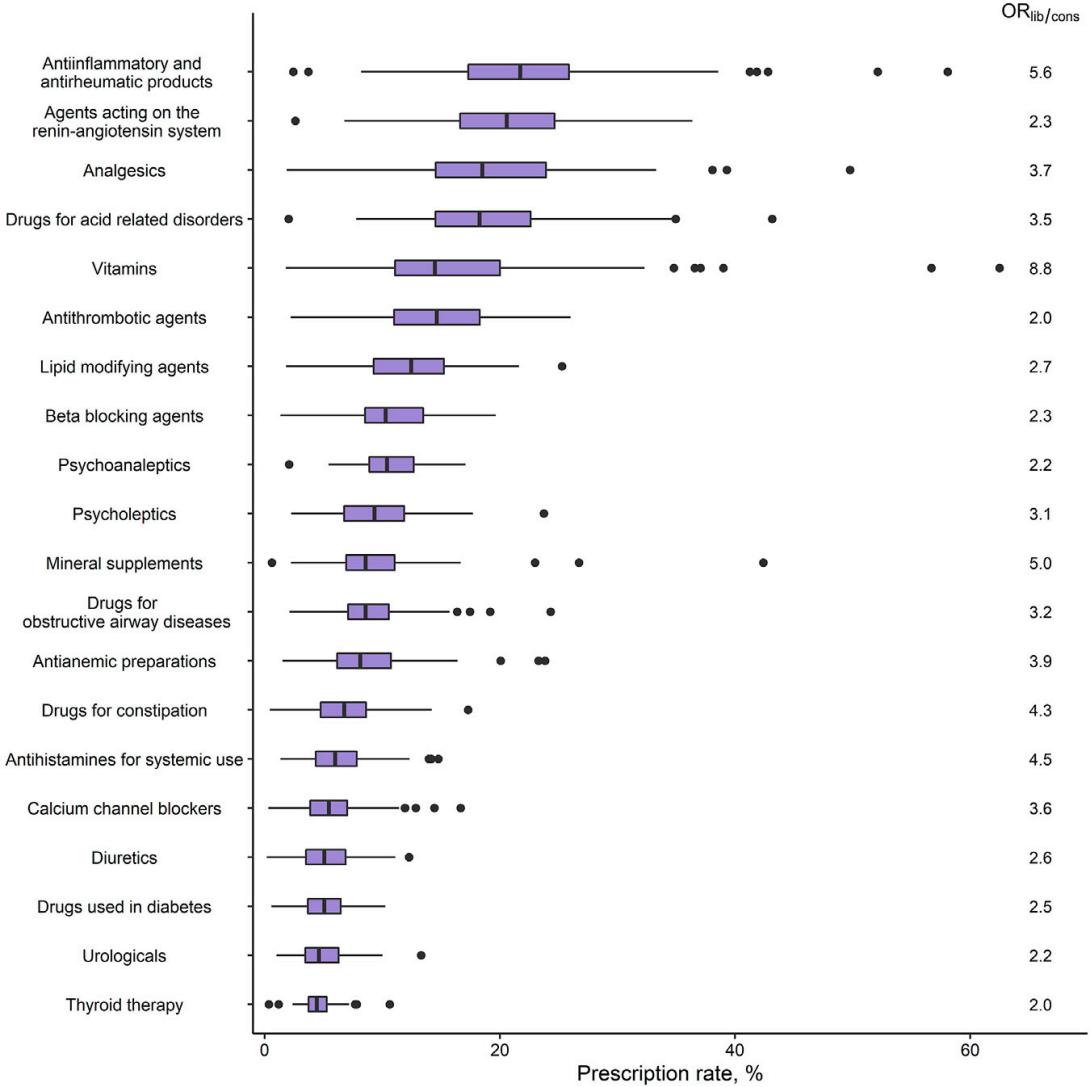
Practitioner variability in medication class prescribing (GPs: *n* = 100). Boxplots of the crude among-GP distributions of prescription rates, and the unexplained variability in terms of OR_lib/cons_, for the 20 most common medication classes (second level of the anatomical therapeutic chemical classification system) ordered by overall prescription rate. Prescription rates were calculated for each GP as the percentage of their patients who had a prescription within the respective medication class; OR_lib/cons_ represents the OR between a liberal prescriber (95th percentile of the random effects distribution) and a conservative prescriber (5th percentile). Abbreviations: GP, general practitioner; OR, odds ratio.

For polypharmacy, practitioner variability in terms of OR_lib/cons_ was 4.4, meaning that patients of a given sex and age had 4.4 higher odds of having polypharmacy if they were treated by a liberal prescriber compared to a conservative prescriber. Among the medication classes, the highest practitioner variability was observed for *vitamins* (OR_lib/cons_ = 8.8), followed by *antiinflammatory and antirheumatic products* (OR_lib/cons_ = 5.6) and *mineral supplements* (OR_lib/cons_ = 5.0). Lowest variability was observed for *antithrombotic agents* and *thyroid therapy* (both OR_lib/cons_ = 2.0), followed by *psychoanaleptics* and *urologicals* (both OR_lib/cons_ = 2.2). The crude practitioner variabilities of the medication classes (boxplots) along with the OR_lib/cons_ are shown in Figure 4.

## Discussion

Comprehensive displays of medication prescribing are scarce. Our study, designed to fill this gap, confirms patient age and sex differences in prescribing of specific medication classes and polypharmacy and reveals considerable practitioner variability in prescribing. In addition to providing epidemiologic insight, our findings uncover medication classes with high prescription rates and practitioner variability, thereby highlighting potential for improvement.

Overall, we found a polypharmacy prevalence of about 24% in the adult Swiss general practice population, increasing with age from 6% in patients aged 18–40 years to 65% in patients aged 81–92 years. Assessments of polypharmacy in younger individuals are scarce. In this context, our polypharmacy prevalence of 20% in patients aged 41–64 years, consistent with findings from Scotland (Guthrie et al., 2015a), raises concerns that polypharmacy may be an important but underappreciated issue in this age group. For older patients, our findings are in line with those of two recent Swiss studies conducted in similar settings but with fewer GPs and patients (polypharmacy prevalence of 37% in a sample aged 75–80 years and of 60% in a sample aged over 75 years, respectively) (Aubert et al., 2016; Schnegg et al., 2020), as well as with general practice prescribing data from Scotland (Guthrie et al., 2015a). Moreover, two Swiss studies with a population-based sampling strategy and presumably less morbid patients than in our general practice-based study found lower polypharmacy rates compared to our results, as would be expected (Castioni et al., 2017; Schneider et al., 2019). With regard to prescription rates of specific medication classes, we found highest rates for pain and inflammation medication, medication for cardiovascular risk management, and medications to regulate gastric acidity. These prescription rates are highly concordant with those found in similar national (Schnegg et al., 2020; Muheim et al., 2021) and international (Guthrie et al., 2015a) studies.

Our study provides relevant insights into the relationship between prescription rates and patient demographics. For age, we found a positive association with prescription rates for most medications, which we expected given the accumulation of chronic diseases with age. However, prescription rates of *lipid modifying agents* decreased markedly beyond 80 years of age in both sexes. This finding might be the result of many GPs’ willingness to de-prescribe these medications for patients in primary prevention of cardiovascular diseases (Jungo et al., 2021). Similarly, the prescription rates of *antiinflammatory and antirheumatic products* decreased in the older. It is quite plausible that this is also partly due to de-prescribing, as there is a broad consensus that these drugs are potentially inappropriate for older patients (Holt et al., 2010; Panel et al., 2019). In this context, it is worth noting that we found high rates of *analgesics* in the oldest age group. One might thus hypothesize that antiinflammatory drugs are replaced by analgesics (mostly paracetamol) in the old, especially considering that pain was the most frequent complaint in a study of old, multimorbid patients in Swiss general practice (Neuner-Jehle et al., 2017). On another note, however, the age-dependent increase in the prescription rates of *diuretics* also in the very old population is of particular concern, given their association with preventable hospitalizations (Howard et al., 2007).

Regarding sex differences, we found a higher rate of polypharmacy in women, consistent with the literature (Guthrie et al., 2015a; Schnegg et al., 2020). The difference was more pronounced at younger ages, and interestingly, hormonal contraceptives did not relevantly contribute to the difference. The most decisive drivers of the differing rates in the younger population seemed to be higher prescription rates of *vitamins*, *mineral supplements*, and *antianemic preparations* in female patients. Physiological reasons may explain the higher prescription rates of *vitamins* for women (i.e., folic acid for the prevention of embryonal neural tube defects during reproductive age, or vitamin D to prevent osteoporosis at advanced age). For *mineral supplements*, the sex difference at older ages may also be partly explained by physiology (i.e., with calcium supplementation for the prevention of osteoporosis), but this explanation does not readily apply to younger patients. For *antianemic preparations*, prescription rates are higher among women only during reproductive age, which is quite plausibly explained by iron deficiency caused by menstrual blood loss (Benson et al., 2021). Contrary, a higher prescription rate among male patients was observed for cardiovascular medications. While this finding is highly consistent with reports from other studies (Schnegg et al., 2020; Zhao et al., 2020), the higher intrinsic cardiovascular risk of male patients may not be the only explanation, as female patients have been found to receive less intensive cardiovascular care than men even when at similar risk (Rachamin et al., 2020a, 2020b, 2021).

Variability in prescribing can partly be explained by the GPs’ patient populations (case mix). However, it can also hint at (in)appropriateness because some of the variability may be due to medication over- (or under-)use, and the risk of prescribing potentially inappropriate medications is higher among physicians with a more liberal attitude towards prescribing. (Martinez et al., 2021). Hence, several studies have investigated practitioner variability in polypharmacy: A study from Swedish general practice found that the prevalence of polypharmacy varied by a factor of six among all GPs, whereas studies from Germany and the Netherlands found factors of 3.6 and 2.4, respectively (Bjerrum et al., 1999; Grimmsmann and Himmel, 2009; Sinnige et al., 2016). These numbers are, however, sensitive to outliers and therefore of limited informative value. We quelled the influence of outliers by introducing the OR_lib/cons_, which represents the central 90% of GPs. Our result of an OR_lib/cons_ of 4.4 for polypharmacy is therefore a more conservative measure but still illustrates a large practitioner variability, suggesting that there may be much room for improvements in quality of care and potential cost savings.

Medication classes with both high overall prescription rates as well as high OR_lib/cons_ are arguably of particular relevance and include *vitamins*, *mineral supplements*, *antiinflammatory and antirheumatic products*, *antianemic preparations*, and *antihistamins for systemic use*. The highest practitioner variability was found for *vitamins* which is—to our best knowledge—a novel finding. The high practitioner variability in vitamin prescribing is however in line with the high practitioner variability in vitamin testing observed both in Switzerland and internationally (Schumacher et al., 2020; O'Sullivan et al., 2018). Moreover, inappropriate prescribing of vitamins has been documented before, and in Switzerland specifically, a potential overuse of *antianemic preparations* has been suspected (Silverstein et al., 2019; Biétry et al., 2017; Meier et al., 2019). Interestingly, *vitamins*, *mineral supplements*, and *antianemic preparations* were also among those medications which were more often prescribed to (especially young) female patients. For *antiinflammatory and antirheumatic products,* high practitioner variability in prescribing has been acknowledged before, and is most relevant because of the well-known associated risks (Hawkey et al., 1997; Dreischulte et al., 2012). Furthermore, a medication class for which variability and potential overuse have often been investigated, due to their contribution to the emerging threat of multiresistant germs, are *antibacterials*. A recent Italian primary care-based study which investigated prescribing of a set of six frequently prescribed medications found the largest variability in antibiotics (Russo et al., 2020). In our study, antibiotics had a rather low prevalence (and are therefore not described in the main text), but practitioner variability in prescribing antibacterial medication was considerable (OR_lib/cons_ = 4.9, Supplementary Table S2). In contrast, low practitioner variability was found in thyroid medications, antithrombotics, renin-angiotensin antagonists, beta blockers, and antidiabetic drugs. This consistent prescription behavior by GPs is reassuring especially in antithrombotic medication which convey high bleeding risks and require adherence to evidence-based treatment guidelines (Hutten et al., 1999).

### Strengths and Limitations

Strengths of this study lie in the comprehensiveness of medication classes assessed and of the population included, which together provide a large and detailed picture of medication prescribing activity in Swiss general practice. Moreover, we excluded topical medication in order to increase the relevance of our findings regarding polypharmacy. The data presented in this article is valuable for researchers as well as for policy makers and may help to inform assumptions, make comparisons, and set policy priorities. A limitation of this study are the unknown and potentially varying morbidities of the GPs’ patient populations, which made it impossible to more precisely judge the appropriateness of prescribed medications. In addition, due to the cross-sectional design of our study, we could not follow up prescription dynamics and were therefore unable to distinguish between cautious prescribing and secondary de-prescribing. Moreover, since we analyzed patients at their last visits to their GPs in 2019, we measured prescriptions immediately following a medical consultation and our results are therefore bound to an episode of care which may not be fully representative for a full patient year and may have overestimated prescription rates. This limitation would primarily affect drugs prescribed for acute indications, such as *analgesics* and *antiinflammatory and antirheumatic products*. Analyzing data from last encounters in 2019 also caused an overrepresentation of the winter season and may have biased prescription rates for medication targeting seasonal illnesses, e.g., antibiotics (Hawes et al., 2018). Lastly, we did not consider prescribed medication doses, which is also a limitation for judging adequacy of prescriptions.

## Conclusion

Based on practitioner variability, prevalence and conveyed risks, the targets with the highest potential for subsequent initiatives to improve medication prescribing in Swiss general practice are antiinflammatory medications and polypharmacy in old and very old patients.

## Data Availability

The datasets presented in this article are not readily available because of institutional restrictions. Requests to access the datasets should be directed to thomas.rosemann@usz.ch.

## References

[B1] Aubert C. E. Streit S. Da Costa B. R. Collet T. H. Cornuz J. Gaspoz J. M. (2016). Polypharmacy and Specific Comorbidities in university Primary Care Settings. Eur. J. Intern. Med. 35, 35–42. 10.1016/j.ejim.2016.05.022 27289492

[B2] Barnett K. Mercer S. W. Norbury M. Watt G. Wyke S. Guthrie B. (2012). Epidemiology of Multimorbidity and Implications for Health Care, Research, and Medical Education: a Cross-Sectional Study. Lancet 380 (9836), 37–43. 10.1016/S0140-6736(12)60240-2 22579043

[B3] Benjamin D. J. Berger J. O. Johannesson M. Nosek B. A. Wagenmakers E. J. Berk R. (2018). Redefine Statistical Significance. Nat. Hum. Behav. 2 (1), 6–10. 10.1038/s41562-017-0189-z 30980045

[B4] Benson C. S. Shah A. Stanworth S. J. Frise C. J. Spiby H. Lax S. J. (2021). The Effect of Iron Deficiency and Anaemia on Women's Health. Anaesthesia 76 (S4), 84–95. 10.1111/anae.15405 33682105

[B5] Biétry F. A. Hug B. Reich O. Susan J. S. Meier C. R. (2017). Iron Supplementation in Switzerland - A Bi-national, Descriptive and Observational Study. Swiss Med. Wkly 147, w14444. 10.4414/smw.2017.14444 28695564

[B6] Bjerrum L. Søgaard J. Hallas J. Kragstrup J. (1999). Polypharmacy in General Practice: Differences between Practitioners. Br. J. Gen. Pract. 49 (440), 195–198. 10343422 PMC1313371

[B7] Cao X. Hou Y. Zhang X. Xu C. Jia P. Sun X. (2020). A Comparative, Correlate Analysis and Projection of Global and Regional Life Expectancy, Healthy Life Expectancy, and Their GAP: 1995-2025. J. Glob. Health 10 (2), 020407. 10.7189/jogh.10.020407 33110572 PMC7568920

[B8] Castioni J. Marques-Vidal P. Abolhassani N. Vollenweider P. Waeber G. (2017). Prevalence and Determinants of Polypharmacy in Switzerland: Data from the CoLaus Study. BMC Health Serv. Res. 17 (1), 840. 10.1186/s12913-017-2793-z 29268737 PMC5740765

[B9] Chmiel C. Bhend H. Senn O. Zoller M. Rosemann T. (2011). The FIRE Project: a Milestone for Research in Primary Care in Switzerland. Swiss Med. Wkly 140, w13142. 10.4414/smw.2011.13142 21279858

[B11] Coyle R. Feher M. Jones S. Hamilton M. de Lusignan S. (2019). Variation in the Diagnosis and Control of Hypertension Is Not Explained by Conventional Variables: Cross-Sectional Database Study in English General Practice. PLoS One 14 (1), e0210657. 10.1371/journal.pone.0210657 30629703 PMC6328229

[B12] Donaldson L. J. Kelley E. T. Dhingra-Kumar N. Kieny M. P. Sheikh A. (2017). Medication without Harm: WHO's Third Global Patient Safety Challenge. Lancet 389 (10080), 1680–1681. 10.1016/S0140-6736(17)31047-4 28463129

[B13] Dreischulte T. Grant A. Donnan P. McCowan C. Davey P. Petrie D. (2012). A Cluster Randomised Stepped Wedge Trial to Evaluate the Effectiveness of a Multifaceted Information Technology-Based Intervention in Reducing High-Risk Prescribing of Non-steroidal Anti-inflammatory Drugs and Antiplatelets in Primary Medical Care: the DQIP Study Protocol. Implement Sci. 7, 24. 10.1186/1748-5908-7-24 22444945 PMC3353207

[B10] GBD 2017 Mortality Collaborators (2018). Global, Regional, and National Age-sex-specific Mortality and Life Expectancy, 1950-2017: a Systematic Analysis for the Global Burden of Disease Study 2017. Lancet 392 (10159), 1684–1735. 10.1016/S0140-6736(18)31891-9 30496102 PMC6227504

[B14] Grimmsmann T. Himmel W. (2009). Polypharmacy in Primary Care Practices: an Analysis Using a Large Health Insurance Database. Pharmacoepidemiol. Drug Saf. 18 (12), 1206–1213. 10.1002/pds.1841 19795368

[B15] Guthrie B. Makubate B. Hernandez-Santiago V. Dreischulte T. (2015). The Rising Tide of Polypharmacy and Drug-Drug Interactions: Population Database Analysis 1995-2010. BMC Med. 13 (1), 74. 10.1186/s12916-015-0322-7 25889849 PMC4417329

[B16] Guthrie B. Yu N. Murphy D. Donnan P. T. Dreischulte T. (2015). Measuring Prevalence, Reliability and Variation in High-Risk Prescribing in General Practice Using Multilevel Modelling of Observational Data in a Population Database. Southampton, UK: NIHR Journals Library. 26539601

[B17] Haastrup P. F. Rasmussen S. Hansen J. M. Christensen R. D. Søndergaard J. Jarbøl D. E. (2016). General Practice Variation when Initiating Long-Term Prescribing of Proton Pump Inhibitors: a Nationwide Cohort Study. BMC Fam. Pract. 17, 57. 10.1186/s12875-016-0460-9 27233634 PMC4884377

[B18] Hawes L. Turner L. Buising K. Mazza D. (2018). Use of Electronic Medical Records to Describe General Practitioner Antibiotic Prescribing Patterns. Aust. J. Gen. Pract. 47 (11), 796–800. 10.31128/AJGP-05-18-4570 31207679

[B19] Hawkey C. J. Cullen D. J. Greenwood D. C. Wilson J. V. Logan R. F. (1997). Prescribing of Nonsteroidal Anti-inflammatory Drugs in General Practice: Determinants and Consequences. Aliment. Pharmacol. Ther. 11 (2), 293–298. 10.1046/j.1365-2036.1997.150326000.x 9146765

[B20] Holt S. Schmiedl S. Thürmann P. A. (2010). Potentially Inappropriate Medications in the Elderly: the PRISCUS List. Dtsch Arztebl Int. 107 (31-32), 543–551. 10.3238/arztebl.2010.0543 20827352 PMC2933536

[B21] Howard R. L. Avery A. J. Slavenburg S. Royal S. Pipe G. Lucassen P. (2007). Which Drugs Cause Preventable Admissions to Hospital? A Systematic Review. Br. J. Clin. Pharmacol. 63 (2), 136–147. 10.1111/j.1365-2125.2006.02698.x 16803468 PMC2000562

[B22] Hutten B. A. Lensing A. W. Kraaijenhagen R. A. Prins M. H. (1999). Safety of Treatment with Oral Anticoagulants in the Elderly. A Systematic Review. Drugs Aging 14 (4), 303–312. 10.2165/00002512-199914040-00005 10319243

[B23] Insani W. N. Whittlesea C. Alwafi H. Man K. K. C. Chapman S. Wei L. (2021). Prevalence of Adverse Drug Reactions in the Primary Care Setting: A Systematic Review and Meta-Analysis. PLoS One 16 (5), e0252161. 10.1371/journal.pone.0252161 34038474 PMC8153435

[B24] Jungo K. T. Mantelli S. Rozsnyai Z. Missiou A. Kitanovska B. G. Weltermann B. (2021). General Practitioners' Deprescribing Decisions in Older Adults with Polypharmacy: a Case Vignette Study in 31 Countries. BMC Geriatr. 21 (1), 19. 10.1186/s12877-020-01953-6 33413142 PMC7792080

[B25] Khezrian M. McNeil C. J. Murray A. D. Myint P. K. (2020). An Overview of Prevalence, Determinants and Health Outcomes of Polypharmacy. Ther. Adv. Drug Saf. 11, 2042098620933741. 10.1177/2042098620933741 32587680 PMC7294476

[B26] Kongkaew C. Hann M. Mandal J. Williams S. D. Metcalfe D. Noyce P. R. (2013). Risk Factors for Hospital Admissions Associated with Adverse Drug Events. Pharmacotherapy 33 (8), 827–837. 10.1002/phar.1287 23686895

[B27] Martinez K. A. Linfield D. T. Gupta N. M. Alapati M. V. Moussa D. Hu B. (2021). Patient and Physician Factors Contributing to Polypharmacy Among Older Patients. Curr. Med. Res. Opin. 38, 1–8. 10.1080/03007995.2021.1982683 34544289

[B28] McGettigan P. Henry D. (2011). Cardiovascular Risk with Non-steroidal Anti-inflammatory Drugs: Systematic Review of Population-Based Controlled Observational Studies. Plos Med. 8 (9), e1001098. 10.1371/journal.pmed.1001098 21980265 PMC3181230

[B29] Meier R. Keizer E. Rosemann T. Markun S. (2019). Indications and Associated Factors for Prescribing Intravenous Iron Supplementation in Swiss General Practice: a Retrospective Observational Study. Swiss Med. Wkly 149, w20127. 10.4414/smw.2019.20127 31568551

[B30] mfe Haus- und Kinderärzte Schweiz (2020). Hausärztemangel – aber mit Licht am Horizont: Workforce-Studie 2020 – das Wichtigste in Kürze 2020. Available at: https://www.mfe-standpunkte.ch/de/ausgabe/ausgabe-22020--43/artikel/hausaerztemangel-aber-mit-licht-am-horizont--76 .

[B31] Midão L. Giardini A. Menditto E. Kardas P. Costa E. (2018). Polypharmacy Prevalence Among Older Adults Based on the Survey of Health, Ageing and Retirement in Europe. Arch. Gerontol. Geriatr. 78, 213–220. 10.1016/j.archger.2018.06.018 30015057

[B32] Muheim L. Signorell A. Markun S. Chmiel C. Neuner-Jehle S. Blozik E. (2021). Potentially Inappropriate Proton-Pump Inhibitor Prescription in the General Population: a Claims-Based Retrospective Time Trend Analysis. Ther. Adv. Drug Saf. 14, 1756284821998928. 10.1177/1756284821998928 PMC8053831 33948109

[B33] Neuner-Jehle S. Zechmann S. Grundmann Maissen D. Rosemann T. Senn O. (2017). Patient-provider Concordance in the Perception of Illness and Disease: a Cross-Sectional Study Among Multimorbid Patients and Their General Practitioners in Switzerland. Patient Prefer Adherence 11, 1451–1458. 10.2147/PPA.S137388 28860728 PMC5572955

[B34] O'Sullivan J. W. Stevens S. Oke J. Hobbs F. D. R. Salisbury C. Little P. (2018). Practice Variation in the Use of Tests in UK Primary Care: a Retrospective Analysis of 16 Million Tests Performed over 3.3 Million Patient Years in 2015/16. BMC Med. 16 (1), 229. 10.1186/s12916-018-1217-1 30567539 PMC6300913

[B35] Panel A. G. S. B. C. U. E. Fick D. M. Semla T. P. Steinman M. Beizer J. Brandt N. (2019). American Geriatrics Society 2019 Updated AGS Beers Criteria® for Potentially Inappropriate Medication Use in Older Adults. J. Am. Geriatr. Soc. 67 (4), 674–694. 10.1111/jgs.15767 30693946

[B36] R Core Team. R (2019). A Language and Environment for Statistical Computing. Vienna, Austria: R Foundation for Statistical Computing.

[B37] Rachamin Y. Grischott T. Rosemann T. Meyer M. R. (2021). Inferior Control of Low-Density Lipoprotein Cholesterol in Women Is the Primary Sex Difference in Modifiable Cardiovascular Risk: A Large-Scale, Cross-Sectional Study in Primary Care. Atherosclerosis 324, 141–147. 10.1016/j.atherosclerosis.2021.02.024 33810858

[B38] Rachamin Y. Markun S. Grischott T. Rosemann T. Meier R. (2020). Guideline Concordance of Statin Treatment Decisions: A Retrospective Cohort Study. J. Clin. Med. 9 (11), 3719. 10.3390/jcm9113719 PMC7699602 33228169

[B39] Rachamin Y. Meier R. Rosemann T. Langenegger S. Markun S. (2020). Statin Treatment and LDL Target Value Achievement in Swiss General Practice - a Retrospective Observational Study. Swiss Med. Wkly 150, w20244. 10.4414/smw.2020.20244 32459361

[B40] Russo V. Orlando V. Monetti V. M. Galimberti F. Casula M. Olmastroni E. (2020). Geographical Variation in Medication Prescriptions: A Multiregional Drug-Utilization Study. Front. Pharmacol. 11, 418. 10.3389/fphar.2020.00418 32536861 PMC7269055

[B41] Schnegg D. Senn N. Bugnon O. Schwarz J. Mueller Y. (2020). Drug Prescription in Older Swiss Men and Women Followed in Family Medicine. Drugs Real World Outcomes 7 (1), 87–95. 10.1007/s40801-019-00175-6 31845213 PMC7060976

[B42] Schneider R. Reinau D. Schur N. Blozik E. Früh M. Signorell A. (2019). Drug Prescription Patterns, Polypharmacy and Potentially Inappropriate Medication in Swiss Nursing Homes: a Descriptive Analysis Based on Claims Data. Swiss Med. Wkly 149, w20126. 10.4414/smw.2019.20126 31568557

[B43] Schumacher L. D. Jäger L. Meier R. Rachamin Y. Senn O. Rosemann T. (2020). Trends and Between-Physician Variation in Laboratory Testing: A Retrospective Longitudinal Study in General Practice. J. Clin. Med. 9 (6), 1787. 10.3390/jcm9061787 PMC7355885 32521786

[B44] Silverstein W. K. Lin Y. Dharma C. Croxford R. Earle C. C. Cheung M. C. (2019). Prevalence of Inappropriateness of Parenteral Vitamin B12 Administration in Ontario, Canada. JAMA Intern. Med. 179 (10), 1434–1436. 10.1001/jamainternmed.2019.1859 31305876 PMC6632124

[B45] Singh G. Triadafilopoulos G. (1999). Epidemiology of NSAID Induced Gastrointestinal Complications. J. Rheumatol. Suppl. 56, 18–24. 10225536

[B46] Sinnige J. Braspenning J. C. Schellevis F. G. Hek K. Stirbu I. Westert G. P. (2016). Inter-practice Variation in Polypharmacy Prevalence Amongst Older Patients in Primary Care. Pharmacoepidemiol. Drug Saf. 25 (9), 1033–1041. 10.1002/pds.4016 27133740

[B47] Wennberg J. E. (2011). Time to Tackle Unwarranted Variations in Practice. Bmj 342, d1513. 10.1136/bmj.d1513 21415111

[B48] WHOCC (2020). ATC/DDD Index 2020: WHO Collaborating Centre for Drug Statistics Methodology. Available at: https://www.whocc.no/atc_ddd_index/ .

[B49] Zhao M. Woodward M. Vaartjes I. Millett E. R. C. Klipstein-Grobusch K. Hyun K. (2020). Sex Differences in Cardiovascular Medication Prescription in Primary Care: A Systematic Review and Meta-Analysis. J. Am. Heart Assoc. 9 (11), e014742. 10.1161/JAHA.119.014742 32431190 PMC7429003

