# Supplementary material for: Prescription Rates, Polypharmacy and Prescriber Variability in Swiss General Practice—A Cross-Sectional Database Study
Source: Front Pharmacol. 2022 Feb 14;13:832994. doi: 10.3389/fphar.2022.832994 (PMC8884695; doi:10.3389/fphar.2022.832994)
Supplement: Supplementary file 1 [file DataSheet1.pdf]

Supplementary Material

Supplementary Table S1. Description of patients (*n* = 111 811) by sex

**Variables**	**Female (*n* = 57 852)**	**Male (*n* = 53 959)**
Age, median (IQR) [years]	52 (35-68)	52 (36-66)
Number of consultations in 2019, median (IQR)	4 (2-9)	4 (2-8)
Medication count, median (IQR)	2 (0-5)	1 (0-4)
Prevalence of polypharmacy (5 or more medications), %	26.2	21.6
Prescription rates, %		
Antiinflammatory and antirheumatic products	23.7	19.3
Agents acting on the renin-angiotensin system	17.4	22.5
Analgesics	22.0	15.3
Drugs for acid related disorders	19.0	17.6
Vitamins	20.3	10.4
Antithrombotic agents	11.7	16.9
Lipid-modifying agents	9.1	15.4
Beta blocking agents	10.2	10.9
Psychoanaleptics	12.6	8.2
Psycholeptics	11.2	7.3
Mineral supplements	11.4	6.1
Drugs for obstructive airway diseases	9.1	8.3
Antianemic preparations	11.4	5.1
Drugs for constipation	7.8	5.5
Antihistamines for systemic use	7.0	4.9

Calcium channel blockers	5.6	5.3
Diuretics	5.4	4.9
Drugs used in diabetes	4.0	6.1
Urologicals	1.4	8.8
Thyroid therapy	7.0	1.7

Abbreviations: IQR, interquartile range

Supplementary Table S2. Results of regression analyses. Restricted to medication classes with prescription rates >1%.

**Independent variables**	**OR**	**99.5% CI**	***p*-value**
**Polypharmacy ($OR_{lib/cons}$ = 4.42)**			
For female patients (ref.: 18-40 years)			
Age group 41-64 years	3.07	2.80 to 3.36	<0.001
Age group 65-80 years	9.86	8.97 to 10.85	<0.001
Age group 81-92 years	24.04	21.38 to 27.03	<0.001
For male patients (ref.: 18-40 years)			
Age group 41-64 years	4.94	4.39 to 5.56	<0.001
Age group 65-80 years	19.09	16.91 to 21.56	<0.001
Age group 81-92 years	40.83	35.15 to 47.42	<0.001
For patients aged 18-40 years: Male sex (ref: female)	0.49	0.43 to 0.55	<0.001
For patients aged 41-64 years: Male sex (ref: female)	0.78	0.73 to 0.84	<0.001
For patients aged 65-80 years: Male sex (ref: female)	0.94	0.87 to 1.02	0.024
For patients aged 81-92 years: Male sex (ref: female)	0.82	0.72 to 0.94	<0.001
**Antiinflammatory and antirheumatic products ($OR_{lib/cons}$ = 5.60)**			
For female patients (ref.: 18-40 years)			
Age group 41-64 years	1.78	1.65 to 1.91	<0.001
Age group 65-80 years	2.02	1.86 to 2.20	<0.001
Age group 81-92 years	1.17	1.04 to 1.32	<0.001

For male patients (ref.: 18-40 years)			
Age group 41-64 years	1.43	1.32 to 1.54	<0.001
Age group 65-80 years	1.50	1.37 to 1.65	<0.001
Age group 81-92 years	1.00	0.86 to 1.15	0.931
For patients aged 18-40 years: Male sex (ref: female)	0.89	0.82 to 0.97	<0.001
For patients aged 41-64 years: Male sex (ref: female)	0.72	0.67 to 0.76	<0.001
For patients aged 65-80 years: Male sex (ref: female)	0.66	0.61 to 0.72	<0.001
For patients aged 81-92 years: Male sex (ref: female)	0.76	0.64 to 0.89	<0.001
**Agents acting on the renin-angiotensin system ($OR_{lib/cons}$ = 2.29)**			
For female patients (ref.: 18-40 years)			
Age group 41-64 years	21.59	16.83 to 27.70	<0.001
Age group 65-80 years	81.56	63.56 to 104.66	<0.001
Age group 81-92 years	121.71	94.24 to 157.19	<0.001
For male patients (ref.: 18-40 years)			
Age group 41-64 years	14.85	12.61 to 17.48	<0.001
Age group 65-80 years	44.92	38.04 to 53.05	<0.001
Age group 81-92 years	47.32	39.30 to 56.98	<0.001
For patients aged 18-40 years: Male sex (ref: female)	2.66	1.99 to 3.55	<0.001
For patients aged 41-64 years: Male sex (ref: female)	1.83	1.70 to 1.97	<0.001

For patients aged 65-80 years: Male sex (ref: female)	1.47	1.36 to 1.58	<0.001
For patients aged 81-92 years: Male sex (ref: female)	1.03	0.91 to 1.18	0.451
**Analgesics ($OR_{lib/cons}$ = 3.69)**			
For female patients (ref.: 18-40 years)			
Age group 41-64 years	1.69	1.56 to 1.83	<0.001
Age group 65-80 years	2.62	2.40 to 2.86	<0.001
Age group 81-92 years	6.85	6.17 to 7.61	<0.001
For male patients (ref.: 18-40 years)			
Age group 41-64 years	1.55	1.42 to 1.70	<0.001
Age group 65-80 years	2.28	2.06 to 2.53	<0.001
Age group 81-92 years	4.83	4.24 to 5.49	<0.001
For patients aged 18-40 years: Male sex (ref: female)	0.73	0.66 to 0.80	<0.001
For patients aged 41-64 years: Male sex (ref: female)	0.67	0.62 to 0.72	<0.001
For patients aged 65-80 years: Male sex (ref: female)	0.63	0.58 to 0.69	<0.001
For patients aged 81-92 years: Male sex (ref: female)	0.51	0.45 to 0.58	<0.001
**Drugs for acid related disorders ($OR_{lib/cons}$ = 3.46)**			
For female patients (ref.: 18-40 years)			
Age group 41-64 years	2.68	2.45 to 2.94	<0.001
Age group 65-80 years	5.00	4.53 to 5.51	<0.001

Age group 81-92 years	6.38	5.68 to 7.17	<0.001
For male patients (ref.: 18-40 years)			
Age group 41-64 years	2.56	2.33 to 2.82	<0.001
Age group 65-80 years	4.55	4.12 to 5.04	<0.001
Age group 81-92 years	5.23	4.57 to 5.98	<0.001
For patients aged 18-40 years: Male sex (ref: female)	0.99	0.89 to 1.11	0.847
For patients aged 41-64 years: Male sex (ref: female)	0.95	0.88 to 1.02	0.036
For patients aged 65-80 years: Male sex (ref: female)	0.90	0.83 to 0.98	<0.001
For patients aged 81-92 years: Male sex (ref: female)	0.81	0.71 to 0.93	<0.001
**Vitamins ($OR_{lib/cons}$ = 8.80)**			
For female patients (ref.: 18-40 years)			
Age group 41-64 years	2.33	2.13 to 2.55	<0.001
Age group 65-80 years	4.66	4.23 to 5.13	<0.001
Age group 81-92 years	6.62	5.90 to 7.43	<0.001
For male patients (ref.: 18-40 years)			
Age group 41-64 years	2.41	2.11 to 2.75	<0.001
Age group 65-80 years	5.35	4.67 to 6.12	<0.001
Age group 81-92 years	11.25	9.59 to 13.20	<0.001
For patients aged 18-40 years: Male sex (ref: female)	0.39	0.34 to 0.44	<0.001

For patients aged 41-64 years: Male sex (ref: female)	0.40	0.37 to 0.43	<0.001
For patients aged 65-80 years: Male sex (ref: female)	0.44	0.40 to 0.49	<0.001
For patients aged 81-92 years: Male sex (ref: female)	0.65	0.57 to 0.75	<0.001
**Antithrombotic agents ($OR_{lib/cons}$ = 2.05)**			
For female patients (ref.: 18-40 years)			
Age group 41-64 years	4.20	3.43 to 5.14	<0.001
Age group 65-80 years	24.96	20.57 to 30.28	<0.001
Age group 81-92 years	68.31	55.88 to 83.50	<0.001
For male patients (ref.: 18-40 years)			
Age group 41-64 years	11.58	9.37 to 14.30	<0.001
Age group 65-80 years	58.00	46.98 to 71.60	<0.001
Age group 81-92 years	121.04	96.50 to 151.82	<0.001
For patients aged 18-40 years: Male sex (ref: female)	0.87	0.66 to 1.13	0.135
For patients aged 41-64 years: Male sex (ref: female)	2.39	2.15 to 2.65	<0.001
For patients aged 65-80 years: Male sex (ref: female)	2.01	1.85 to 2.19	<0.001
For patients aged 81-92 years: Male sex (ref: female)	1.53	1.35 to 1.74	<0.001
**Lipid modifying agents ($OR_{lib/cons}$ = 2.66)**			
For female patients (ref.: 18-40 years)			
Age group 41-64 years	29.34	18.19 to 47.33	<0.001

Age group 65-80 years	152.78	94.95 to 245.82	<0.001
Age group 81-92 years	127.16	78.51 to 205.97	<0.001
For male patients (ref.: 18-40 years)			
Age group 41-64 years	36.08	25.73 to 50.60	<0.001
Age group 65-80 years	136.58	97.36 to 191.60	<0.001
Age group 81-92 years	115.61	81.45 to 164.11	<0.001
For patients aged 18-40 years: Male sex (ref: female)	2.07	1.15 to 3.72	<0.001
For patients aged 41-64 years: Male sex (ref: female)	2.54	2.30 to 2.81	<0.001
For patients aged 65-80 years: Male sex (ref: female)	1.85	1.70 to 2.01	<0.001
For patients aged 81-92 years: Male sex (ref: female)	1.88	1.63 to 2.17	<0.001
**Beta blocking agents ($OR_{lib/cons}$ = 2.26)**			
For female patients (ref.: 18-40 years)			
Age group 41-64 years	6.04	4.90 to 7.46	<0.001
Age group 65-80 years	23.57	19.17 to 28.97	<0.001
Age group 81-92 years	43.93	35.45 to 54.43	<0.001
For male patients (ref.: 18-40 years)			
Age group 41-64 years	9.81	7.77 to 12.38	<0.001
Age group 65-80 years	34.79	27.60 to 43.84	<0.001
Age group 81-92 years	52.43	40.99 to 67.07	<0.001

For patients aged 18-40 years: Male sex (ref: female)	0.81	0.61 to 1.09	0.046
For patients aged 41-64 years: Male sex (ref: female)	1.32	1.19 to 1.46	<0.001
For patients aged 65-80 years: Male sex (ref: female)	1.20	1.09 to 1.31	<0.001
For patients aged 81-92 years: Male sex (ref: female)	0.97	0.85 to 1.11	0.507
**Psychoanaleptics ($OR_{lib/cons}$ = 2.23)**			
For female patients (ref.: 18-40 years)			
Age group 41-64 years	2.08	1.88 to 2.30	<0.001
Age group 65-80 years	2.44	2.18 to 2.73	<0.001
Age group 81-92 years	4.24	3.73 to 4.81	<0.001
For male patients (ref.: 18-40 years)			
Age group 41-64 years	1.66	1.48 to 1.86	<0.001
Age group 65-80 years	1.61	1.41 to 1.84	<0.001
Age group 81-92 years	2.79	2.36 to 3.31	<0.001
For patients aged 18-40 years: Male sex (ref: female)	0.81	0.71 to 0.92	<0.001
For patients aged 41-64 years: Male sex (ref: female)	0.64	0.59 to 0.70	<0.001
For patients aged 65-80 years: Male sex (ref: female)	0.53	0.47 to 0.60	<0.001
For patients aged 81-92 years: Male sex (ref: female)	0.53	0.45 to 0.63	<0.001
**Psycholeptics ($OR_{lib/cons}$ = 3.06)**			
For female patients (ref.: 18-40 years)			

Age group 41-64 years	3.22	2.80 to 3.70	<0.001
Age group 65-80 years	6.97	6.05 to 8.02	<0.001
Age group 81-92 years	14.67	12.61 to 17.07	<0.001
For male patients (ref.: 18-40 years)			
Age group 41-64 years	2.20	1.90 to 2.54	<0.001
Age group 65-80 years	3.39	2.91 to 3.95	<0.001
Age group 81-92 years	7.40	6.21 to 8.82	<0.001
For patients aged 18-40 years: Male sex (ref: female)	1.04	0.88 to 1.24	0.502
For patients aged 41-64 years: Male sex (ref: female)	0.71	0.64 to 0.79	<0.001
For patients aged 65-80 years: Male sex (ref: female)	0.51	0.45 to 0.57	<0.001
For patients aged 81-92 years: Male sex (ref: female)	0.53	0.45 to 0.61	<0.001
**Mineral supplements ($OR_{lib/cons}$ = 5.04)**			
For female patients (ref.: 18-40 years)			
Age group 41-64 years	1.79	1.61 to 2.00	<0.001
Age group 65-80 years	3.24	2.88 to 3.64	<0.001
Age group 81-92 years	4.35	3.80 to 4.98	<0.001
For male patients (ref.: 18-40 years)			
Age group 41-64 years	2.21	1.86 to 2.62	<0.001
Age group 65-80 years	5.60	4.72 to 6.64	<0.001
Age group 81-92 years	9.30	7.63 to 11.33	<0.001

For patients aged 18-40 years: Male sex (ref: female)	0.35	0.30 to 0.42	<0.001
For patients aged 41-64 years: Male sex (ref: female)	0.44	0.39 to 0.49	<0.001
For patients aged 65-80 years: Male sex (ref: female)	0.61	0.55 to 0.69	<0.001
For patients aged 81-92 years: Male sex (ref: female)	0.76	0.64 to 0.89	<0.001
**Drugs for obstructive airway diseases (OR$_{lib/cons}$ = 3.20)**			
For female patients (ref.: 18-40 years)			
Age group 41-64 years	1.22	1.10 to 1.35	<0.001
Age group 65-80 years	1.56	1.38 to 1.75	<0.001
Age group 81-92 years	1.43	1.22 to 1.66	<0.001
For male patients (ref.: 18-40 years)			
Age group 41-64 years	1.28	1.14 to 1.43	<0.001
Age group 65-80 years	1.87	1.65 to 2.12	<0.001
Age group 81-92 years	1.99	1.67 to 2.37	<0.001
For patients aged 18-40 years: Male sex (ref: female)	0.82	0.73 to 0.92	<0.001
For patients aged 41-64 years: Male sex (ref: female)	0.86	0.78 to 0.95	<0.001
For patients aged 65-80 years: Male sex (ref: female)	0.99	0.87 to 1.11	0.741
For patients aged 81-92 years: Male sex (ref: female)	1.14	0.93 to 1.39	0.063
**Antianemic preparations (OR$_{lib/cons}$ = 3.86)**			
For female patients (ref.: 18-40 years)			

Age group 41-64 years	0.80	0.73 to 0.88	<0.001
Age group 65-80 years	0.81	0.73 to 0.91	<0.001
Age group 81-92 years	1.62	1.43 to 1.84	<0.001
For male patients (ref.: 18-40 years)			
Age group 41-64 years	2.74	2.22 to 3.38	<0.001
Age group 65-80 years	7.76	6.32 to 9.53	<0.001
Age group 81-92 years	15.49	12.36 to 19.40	<0.001
For patients aged 18-40 years: Male sex (ref: female)	0.10	0.08 to 0.12	<0.001
For patients aged 41-64 years: Male sex (ref: female)	0.34	0.31 to 0.39	<0.001
For patients aged 65-80 years: Male sex (ref: female)	0.96	0.85 to 1.10	0.429
For patients aged 81-92 years: Male sex (ref: female)	0.97	0.82 to 1.14	0.557
**Drugs for constipation ($OR_{lib/cons}$ = 4.31)**			
For female patients (ref.: 18-40 years)			
Age group 41-64 years	1.86	1.60 to 2.15	<0.001
Age group 65-80 years	4.06	3.50 to 4.70	<0.001
Age group 81-92 years	11.06	9.48 to 12.91	<0.001
For male patients (ref.: 18-40 years)			
Age group 41-64 years	2.01	1.66 to 2.44	<0.001
Age group 65-80 years	5.91	4.90 to 7.12	<0.001
Age group 81-92 years	15.42	12.58 to 18.89	<0.001

For patients aged 18-40 years: Male sex (ref: female)	0.57	0.47 to 0.70	<0.001
For patients aged 41-64 years: Male sex (ref: female)	0.62	0.54 to 0.71	<0.001
For patients aged 65-80 years: Male sex (ref: female)	0.83	0.73 to 0.94	<0.001
For patients aged 81-92 years: Male sex (ref: female)	0.79	0.68 to 0.92	<0.001
**Antihistamines for systemic use ($OR_{lib/cons}$ = 4.51)**			
For female patients (ref.: 18-40 years)			
Age group 41-64 years	1.00	0.89 to 1.11	0.920
Age group 65-80 years	0.79	0.68 to 0.90	<0.001
Age group 81-92 years	0.64	0.53 to 0.79	<0.001
For male patients (ref.: 18-40 years)			
Age group 41-64 years	0.76	0.67 to 0.86	<0.001
Age group 65-80 years	0.62	0.53 to 0.74	<0.001
Age group 81-92 years	0.69	0.53 to 0.89	<0.001
For patients aged 18-40 years: Male sex (ref: female)	0.79	0.70 to 0.89	<0.001
For patients aged 41-64 years: Male sex (ref: female)	0.60	0.54 to 0.68	<0.001
For patients aged 65-80 years: Male sex (ref: female)	0.63	0.53 to 0.75	<0.001
For patients aged 81-92 years: Male sex (ref: female)	0.84	0.62 to 1.14	0.112
**Calcium channel blockers ($OR_{lib/cons}$ = 3.57)**			
For female patients (ref.: 18-40 years)			

Age group 41-64 years	13.21	8.38 to 20.84	<0.001
Age group 65-80 years	53.36	34.04 to 83.65	<0.001
Age group 81-92 years	106.79	67.81 to 168.16	<0.001
For male patients (ref.: 18-40 years)			
Age group 41-64 years	14.17	9.64 to 20.84	<0.001
Age group 65-80 years	37.69	25.66 to 55.37	<0.001
Age group 81-92 years	53.16	35.65 to 79.25	<0.001
For patients aged 18-40 years: Male sex (ref: female)	1.34	0.75 to 2.41	0.159
For patients aged 41-64 years: Male sex (ref: female)	1.44	1.25 to 1.66	<0.001
For patients aged 65-80 years: Male sex (ref: female)	0.95	0.84 to 1.07	0.206
For patients aged 81-92 years: Male sex (ref: female)	0.67	0.56 to 0.79	<0.001
**Diuretics ($OR_{lib/cons}$ = 2.63)**			
For female patients (ref.: 18-40 years)			
Age group 41-64 years	13.10	7.35 to 23.33	<0.001
Age group 65-80 years	73.18	41.49 to 129.06	<0.001
Age group 81-92 years	274.13	155.38 to 483.63	<0.001
For male patients (ref.: 18-40 years)			
Age group 41-64 years	20.65	10.84 to 39.34	<0.001
Age group 65-80 years	98.66	52.10 to 186.86	<0.001
Age group 81-92 years	298.41	156.97 to 567.30	<0.001

For patients aged 18-40 years: Male sex (ref: female)	0.80	0.34 to 1.88	0.469
For patients aged 41-64 years: Male sex (ref: female)	1.27	1.05 to 1.53	<0.001
For patients aged 65-80 years: Male sex (ref: female)	1.08	0.96 to 1.23	0.072
For patients aged 81-92 years: Male sex (ref: female)	0.87	0.76 to 1.01	0.008
**Drugs used in diabetes ($OR_{lib/cons}$ = 2.49)**			
For female patients (ref.: 18-40 years)			
Age group 41-64 years	5.70	4.27 to 7.63	<0.001
Age group 65-80 years	15.07	11.31 to 20.08	<0.001
Age group 81-92 years	18.09	13.37 to 24.47	<0.001
For male patients (ref.: 18-40 years)			
Age group 41-64 years	12.83	9.34 to 17.62	<0.001
Age group 65-80 years	32.00	23.29 to 43.95	<0.001
Age group 81-92 years	32.04	22.83 to 44.97	<0.001
For patients aged 18-40 years: Male sex (ref: female)	0.80	0.53 to 1.20	0.120
For patients aged 41-64 years: Male sex (ref: female)	1.80	1.57 to 2.06	<0.001
For patients aged 65-80 years: Male sex (ref: female)	1.70	1.50 to 1.92	<0.001
For patients aged 81-92 years: Male sex (ref: female)	1.42	1.17 to 1.72	<0.001
**Urologicals ($OR_{lib/cons}$ = 2.17)**			
For female patients (ref.: 18-40 years)			

Age group 41-64 years	2.43	1.60 to 3.70	<0.001
Age group 65-80 years	8.04	5.41 to 11.95	<0.001
Age group 81-92 years	14.48	9.64 to 21.75	<0.001
For male patients (ref.: 18-40 years)			
Age group 41-64 years	7.22	5.74 to 9.08	<0.001
Age group 65-80 years	25.26	20.11 to 31.73	<0.001
Age group 81-92 years	32.30	25.24 to 41.33	<0.001
For patients aged 18-40 years: Male sex (ref: female)	3.02	1.99 to 4.60	<0.001
For patients aged 41-64 years: Male sex (ref: female)	8.98	7.16 to 11.26	<0.001
For patients aged 65-80 years: Male sex (ref: female)	9.50	7.96 to 11.34	<0.001
For patients aged 81-92 years: Male sex (ref: female)	6.75	5.41 to 8.41	<0.001
**Thyroid therapy ($OR_{lib/cons}$ = 1.97)**			
For female patients (ref.: 18-40 years)			
Age group 41-64 years	2.33	2.02 to 2.68	<0.001
Age group 65-80 years	3.46	2.98 to 4.01	<0.001
Age group 81-92 years	3.91	3.28 to 4.66	<0.001
For male patients (ref.: 18-40 years)			
Age group 41-64 years	2.95	2.07 to 4.22	<0.001
Age group 65-80 years	5.97	4.18 to 8.54	<0.001
Age group 81-92 years	10.89	7.38 to 16.07	<0.001

For patients aged 18-40 years: Male sex (ref: female)	0.15	0.10 to 0.20	<0.001
For patients aged 41-64 years: Male sex (ref: female)	0.18	0.15 to 0.22	<0.001
For patients aged 65-80 years: Male sex (ref: female)	0.25	0.21 to 0.30	<0.001
For patients aged 81-92 years: Male sex (ref: female)	0.41	0.32 to 0.52	<0.001
**Sex hormones and modulators of the genital system ($OR_{lib/cons}$ = 10.36)**			
For female patients (ref.: 18-40 years)			
Age group 41-64 years	0.59	0.53 to 0.65	<0.001
Age group 65-80 years	0.75	0.67 to 0.85	<0.001
Age group 81-92 years	0.61	0.51 to 0.73	<0.001
For male patients (ref.: 18-40 years)			
Age group 41-64 years	1.72	1.07 to 2.76	0.001
Age group 65-80 years	2.13	1.26 to 3.58	<0.001
Age group 81-92 years	1.31	0.55 to 3.12	0.392
For patients aged 18-40 years: Male sex (ref: female)	0.03	0.02 to 0.04	<0.001
For patients aged 41-64 years: Male sex (ref: female)	0.08	0.06 to 0.10	<0.001
For patients aged 65-80 years: Male sex (ref: female)	0.07	0.05 to 0.10	<0.001
For patients aged 81-92 years: Male sex (ref: female)	0.06	0.02 to 0.12	<0.001
**Antibacterials for systemic use ($OR_{lib/cons}$ = 4.87)**			
For female patients (ref.: 18-40 years)			

Age group 41-64 years	0.76	0.66 to 0.86	<0.001
Age group 65-80 years	0.75	0.64 to 0.88	<0.001
Age group 81-92 years	0.85	0.69 to 1.04	0.027
For male patients (ref.: 18-40 years)			
Age group 41-64 years	0.87	0.74 to 1.02	0.013
Age group 65-80 years	0.98	0.82 to 1.18	0.806
Age group 81-92 years	0.90	0.67 to 1.20	0.300
For patients aged 18-40 years: Male sex (ref: female)	0.61	0.53 to 0.71	<0.001
For patients aged 41-64 years: Male sex (ref: female)	0.71	0.61 to 0.81	<0.001
For patients aged 65-80 years: Male sex (ref: female)	0.80	0.67 to 0.97	0.001
For patients aged 81-92 years: Male sex (ref: female)	0.65	0.47 to 0.89	<0.001
**Cough and cold preparations ($OR_{lib/cons}$ = 7.40)**			
For female patients (ref.: 18-40 years)			
Age group 41-64 years	0.99	0.87 to 1.14	0.914
Age group 65-80 years	1.13	0.96 to 1.32	0.035
Age group 81-92 years	1.06	0.86 to 1.32	0.431
For male patients (ref.: 18-40 years)			
Age group 41-64 years	0.93	0.79 to 1.08	0.165
Age group 65-80 years	1.10	0.92 to 1.32	0.131
Age group 81-92 years	1.38	1.07 to 1.77	<0.001

For patients aged 18-40 years: Male sex (ref: female)	0.81	0.69 to 0.94	<0.001
For patients aged 41-64 years: Male sex (ref: female)	0.75	0.65 to 0.86	<0.001
For patients aged 65-80 years: Male sex (ref: female)	0.79	0.66 to 0.94	<0.001
For patients aged 81-92 years: Male sex (ref: female)	1.05	0.78 to 1.40	0.665
**Drugs for functional gastrointestinal disorders ($OR_{lib/cons}$ = 4.33)**			
For female patients (ref.: 18-40 years)			
Age group 41-64 years	1.05	0.90 to 1.22	0.380
Age group 65-80 years	1.42	1.20 to 1.67	<0.001
Age group 81-92 years	3.00	2.51 to 3.58	<0.001
For male patients (ref.: 18-40 years)			
Age group 41-64 years	0.85	0.70 to 1.03	0.018
Age group 65-80 years	1.15	0.93 to 1.43	0.068
Age group 81-92 years	2.02	1.55 to 2.64	<0.001
For patients aged 18-40 years: Male sex (ref: female)	0.67	0.56 to 0.81	<0.001
For patients aged 41-64 years: Male sex (ref: female)	0.54	0.46 to 0.64	<0.001
For patients aged 65-80 years: Male sex (ref: female)	0.55	0.45 to 0.67	<0.001
For patients aged 81-92 years: Male sex (ref: female)	0.45	0.35 to 0.59	<0.001
**Corticosteroids for systemic use ($OR_{lib/cons}$ = 4.73)**			
For female patients (ref.: 18-40 years)			

Age group 41-64 years	2.00	1.62 to 2.47	<0.001
Age group 65-80 years	3.61	2.91 to 4.48	<0.001
Age group 81-92 years	5.02	3.95 to 6.39	<0.001
For male patients (ref.: 18-40 years)			
Age group 41-64 years	2.10	1.70 to 2.61	<0.001
Age group 65-80 years	3.78	3.03 to 4.72	<0.001
Age group 81-92 years	5.07	3.87 to 6.65	<0.001
For patients aged 18-40 years: Male sex (ref: female)	0.97	0.76 to 1.25	0.762
For patients aged 41-64 years: Male sex (ref: female)	1.02	0.87 to 1.20	0.693
For patients aged 65-80 years: Male sex (ref: female)	1.02	0.86 to 1.21	0.774
For patients aged 81-92 years: Male sex (ref: female)	0.98	0.76 to 1.27	0.850
**Antiepileptics ($OR_{lib/cons}$ = 1.90)**			
For female patients (ref.: 18-40 years)			
Age group 41-64 years	2.10	1.69 to 2.62	<0.001
Age group 65-80 years	3.55	2.84 to 4.44	<0.001
Age group 81-92 years	5.22	4.09 to 6.66	<0.001
For male patients (ref.: 18-40 years)			
Age group 41-64 years	2.16	1.72 to 2.71	<0.001
Age group 65-80 years	3.38	2.66 to 4.29	<0.001
Age group 81-92 years	4.42	3.31 to 5.92	<0.001

For patients aged 18-40 years: Male sex (ref: female)	0.95	0.72 to 1.24	0.571
For patients aged 41-64 years: Male sex (ref: female)	0.97	0.82 to 1.15	0.632
For patients aged 65-80 years: Male sex (ref: female)	0.90	0.75 to 1.08	0.113
For patients aged 81-92 years: Male sex (ref: female)	0.80	0.62 to 1.05	0.021
**Antidiarrheals, intestinal antiinflammatory/antiinfective agents ($OR_{lib/cons}$ = 3.55)**			
For female patients (ref.: 18-40 years)			
Age group 41-64 years	1.04	0.86 to 1.26	0.567
Age group 65-80 years	2.01	1.65 to 2.44	<0.001
Age group 81-92 years	3.09	2.48 to 3.86	<0.001
For male patients (ref.: 18-40 years)			
Age group 41-64 years	0.81	0.66 to 0.98	0.002
Age group 65-80 years	1.12	0.89 to 1.39	0.161
Age group 81-92 years	1.53	1.13 to 2.06	<0.001
For patients aged 18-40 years: Male sex (ref: female)	1.08	0.88 to 1.32	0.295
For patients aged 41-64 years: Male sex (ref: female)	0.84	0.69 to 1.01	0.008
For patients aged 65-80 years: Male sex (ref: female)	0.60	0.48 to 0.74	<0.001
For patients aged 81-92 years: Male sex (ref: female)	0.53	0.39 to 0.72	<0.001
**Cardiac therapy ($OR_{lib/cons}$ = 3.49)**			
For female patients (ref.: 18-40 years)			

Age group 41-64 years	1.39	1.07 to 1.82	<0.001
Age group 65-80 years	4.13	3.22 to 5.30	<0.001
Age group 81-92 years	13.10	10.23 to 16.77	<0.001
For male patients (ref.: 18-40 years)			
Age group 41-64 years	3.25	2.23 to 4.72	<0.001
Age group 65-80 years	14.59	10.22 to 20.83	<0.001
Age group 81-92 years	31.88	22.02 to 46.14	<0.001
For patients aged 18-40 years: Male sex (ref: female)	0.41	0.27 to 0.60	<0.001
For patients aged 41-64 years: Male sex (ref: female)	0.95	0.75 to 1.19	0.498
For patients aged 65-80 years: Male sex (ref: female)	1.43	1.20 to 1.71	<0.001
For patients aged 81-92 years: Male sex (ref: female)	0.99	0.81 to 1.20	0.845
**Muscle relaxants ($OR_{lib/cons}$ = 5.40)**			
For female patients (ref.: 18-40 years)			
Age group 41-64 years	1.66	1.37 to 2.02	<0.001
Age group 65-80 years	1.05	0.82 to 1.35	0.578
Age group 81-92 years	0.65	0.44 to 0.98	0.003
For male patients (ref.: 18-40 years)			
Age group 41-64 years	1.19	0.96 to 1.46	0.020
Age group 65-80 years	0.67	0.50 to 0.90	<0.001
Age group 81-92 years	0.54	0.32 to 0.89	<0.001

For patients aged 18-40 years: Male sex (ref: female)	1.05	0.84 to 1.31	0.544
For patients aged 41-64 years: Male sex (ref: female)	0.75	0.63 to 0.89	<0.001
For patients aged 65-80 years: Male sex (ref: female)	0.67	0.49 to 0.91	<0.001
For patients aged 81-92 years: Male sex (ref: female)	0.86	0.47 to 1.58	0.496
**Drugs for treatment of bone diseases ($OR_{lib/cons}$ = 3.17)**			
For female patients (ref.: 18-40 years)			
Age group 41-64 years	33.12	10.08 to 108.83	<0.001
Age group 65-80 years	173.50	53.12 to 566.73	<0.001
Age group 81-92 years	263.71	80.38 to 865.15	<0.001
For male patients (ref.: 18-40 years)			
Age group 41-64 years	17.79	2.88 to 109.72	<0.001
Age group 65-80 years	54.42	8.88 to 333.33	<0.001
Age group 81-92 years	102.53	16.49 to 637.71	<0.001
For patients aged 18-40 years: Male sex (ref: female)	0.48	0.04 to 5.65	0.405
For patients aged 41-64 years: Male sex (ref: female)	0.26	0.19 to 0.36	<0.001
For patients aged 65-80 years: Male sex (ref: female)	0.15	0.12 to 0.20	<0.001
For patients aged 81-92 years: Male sex (ref: female)	0.19	0.13 to 0.26	<0.001
**Antigout preparations ($OR_{lib/cons}$ = 2.41)**			
For female patients (ref.: 18-40 years)			

Age group 41-64 years	7.81	2.81 to 21.69	<0.001
Age group 65-80 years	43.11	16.14 to 115.17	<0.001
Age group 81-92 years	86.98	32.37 to 233.73	<0.001
For male patients (ref.: 18-40 years)			
Age group 41-64 years	7.51	4.91 to 11.49	<0.001
Age group 65-80 years	24.70	16.24 to 37.56	<0.001
Age group 81-92 years	31.36	20.13 to 48.85	<0.001
For patients aged 18-40 years: Male sex (ref: female)	8.27	2.95 to 23.15	<0.001
For patients aged 41-64 years: Male sex (ref: female)	7.95	5.36 to 11.81	<0.001
For patients aged 65-80 years: Male sex (ref: female)	4.74	3.71 to 6.04	<0.001
For patients aged 81-92 years: Male sex (ref: female)	2.98	2.22 to 4.01	<0.001
**Other nervous system drugs ($OR_{lib/cons}$ = 3.04)**			
For female patients (ref.: 18-40 years)			
Age group 41-64 years	2.66	1.96 to 3.60	<0.001
Age group 65-80 years	4.15	3.03 to 5.67	<0.001
Age group 81-92 years	6.17	4.40 to 8.66	<0.001
For male patients (ref.: 18-40 years)			
Age group 41-64 years	2.81	2.04 to 3.86	<0.001
Age group 65-80 years	2.63	1.84 to 3.76	<0.001
Age group 81-92 years	4.18	2.74 to 6.38	<0.001

For patients aged 18-40 years: Male sex (ref: female)	0.92	0.63 to 1.35	0.544
For patients aged 41-64 years: Male sex (ref: female)	0.97	0.79 to 1.20	0.703
For patients aged 65-80 years: Male sex (ref: female)	0.58	0.44 to 0.77	<0.001
For patients aged 81-92 years: Male sex (ref: female)	0.62	0.43 to 0.91	<0.001
**Immunosuppressants ($OR_{lib/cons}$ = 1.93)**			
For female patients (ref.: 18-40 years)			
Age group 41-64 years	1.82	1.38 to 2.38	<0.001
Age group 65-80 years	2.52	1.89 to 3.36	<0.001
Age group 81-92 years	1.59	1.06 to 2.38	0.001
For male patients (ref.: 18-40 years)			
Age group 41-64 years	1.84	1.35 to 2.51	<0.001
Age group 65-80 years	2.44	1.75 to 3.40	<0.001
Age group 81-92 years	1.66	0.99 to 2.80	0.006
For patients aged 18-40 years: Male sex (ref: female)	0.80	0.57 to 1.13	0.071
For patients aged 41-64 years: Male sex (ref: female)	0.82	0.65 to 1.02	0.012
For patients aged 65-80 years: Male sex (ref: female)	0.78	0.59 to 1.02	0.009
For patients aged 81-92 years: Male sex (ref: female)	0.84	0.48 to 1.48	0.390
**Antiprotozoals ($OR_{lib/cons}$ = 4.97)**			
For female patients (ref.: 18-40 years)			

Age group 41-64 years	1.17	0.91 to 1.50	0.083
Age group 65-80 years	1.20	0.87 to 1.67	0.117
Age group 81-92 years	0.72	0.40 to 1.31	0.125
For male patients (ref.: 18-40 years)			
Age group 41-64 years	0.97	0.73 to 1.30	0.785
Age group 65-80 years	1.05	0.72 to 1.54	0.693
Age group 81-92 years	0.50	0.19 to 1.28	0.038
For patients aged 18-40 years: Male sex (ref: female)	0.77	0.59 to 1.01	0.006
For patients aged 41-64 years: Male sex (ref: female)	0.64	0.49 to 0.84	<0.001
For patients aged 65-80 years: Male sex (ref: female)	0.67	0.45 to 1.02	0.007
For patients aged 81-92 years: Male sex (ref: female)	0.53	0.18 to 1.54	0.093

Abbreviations: OR, odds ratio; CI, confidence interval; $OR_{lib/cons}$, odds of a liberal prescriber divided by the odds of a conservative prescriber

Supplementary Table S3. Prescription rates and ATC codes of medication classes. Restricted to medication classes with prescription rates >1%.

No	Medication class	ATC code (second level)	Prescription rate [%] (total number of patients)
1	Antiinflammatory and antirheumatic products	M01	21.6 (24 147)
2	Agents acting on the renin-angiotensin system	C09	19.9 (22 208)
3	Analgesics	N02	18.7 (20 960)
4	Drugs for acid related disorders	A02	18.3 (20 508)
5	Vitamins	A11	15.5 (17 324)
6	Antithrombotic agents	B01	14.2 (15 887)
7	Lipid modifying agents	C10	12.1 (13 582)
8	Beta blocking agents	C07	10.5 (11 796)
9	Psychoanaleptics	N06	10.5 (11 704)
10	Psycholeptics	N05	9.3 (10 407)
11	Mineral supplements	A12	8.8 (9 894)
12	Drugs for obstructive airway diseases	R03	8.7 (9 734)
13	Antianemic preparations	B03	8.3 (9 334)
14	Drugs for constipation	A06	6.7 (7 480)
15	Antihistamines for systemic use	R06	6.0 (6 700)
16	Calcium channel blockers	C08	5.5 (6 125)
17	Diuretics	C03	5.1 (5 747)
18	Drugs used in diabetes	A10	5.0 (5 641)

19	Urologicals	G04	5.0 (5 541)
20	Thyroid therapy	H03	4.4 (4 946)
21	Sex hormones and modulators of the genital system	G03	4.4 (4 910)
22	Antibacterials for systemic use	J01	4.4 (4 902)
23	Cough and cold preparations	R05	4.3 (4 767)
24	Drugs for functional gastrointestinal disorders	A03	3.5 (3 956)
25	Corticosteroids for systemic use	H02	3.2 (3 537)
26	Antiepileptics	N03	2.9 (3 206)
27	Antidiarrheals, intestinal antiinflammatory/antiinfective agents	A07	2.7 (3 038)
28	Cardiac therapy	C01	2.7 (3 005)
29	Muscle relaxants	M03	2 (2 232)
30	Drugs for treatment of bone diseases	M05	2 (2 215)
31	Antigout preparations	M04	1.7 (1 894)
32	Other nervous system drugs	N07	1.5 (1 696)
33	Immunosuppressants	L04	1.3 (1 486)
34	Antiprotozoals	P01	1.1 (1 195)

Abbreviations: ATC. Anatomical therapeutic chemical
